# 

**Published:** 1891-12

**Authors:** 


EDITED BY

H. V. M. MILLER, M. D., LL. D. | L. P. KENNEDY, A. M., M. D.
VIRGIL O. HARDON, M. D. | M. B. HUTCHINS, M. D.
F. W. McRAE, M. D. | L. B. GRANDY, M. D.

PUBLISHED BY JAS. P. HARRISON & CO., ATLANTA, GA.
Entered at the Post-Office at Atlanta, Ga., as second-class matter.

OLD SERIES VOL. XXV. / NEW SERIES VOL. VIII. } ATLANTA, GA., DECEMBER, 1891. NO. 10.

